# Uterine cervical volume: comparison between two- and three-dimensional ultrasound methods at 20-24 weeks of gestation

**DOI:** 10.1590/0100-3984.2025.0002

**Published:** 2025-08-18

**Authors:** Renan Fonseca Cardozo, Fernando Maia Peixoto-Filho, Edward Araujo Júnior

**Affiliations:** 1 Department of Fetal Medicine, Instituto Nacional de Saúde da Mulher, da Criança e do Adolescente Fernandes Figueira/Fundação Oswaldo Cruz (IFF/Fiocruz), Rio de Janeiro, RJ, Brazil; 2 Department of Obstetrics, Escola Paulista de Medicina da Universidade Federal de São Paulo (EPM-Unifesp), São Paulo, SP, Brazil; 3 Discipline of Women’s Health, Universidade Municipal de São Caetano do Sul (USCS), São Caetano do Sul, SP, Brazil

**Keywords:** Pregnancy, Cervical length measurement, Cervix uteri/embryology, Pregnancy trimester, second, Ultrasonography, prenatal/methods, Reproducibility of results, Gravidez, Medida do comprimento cervical, Colo do útero/embriologia, Segundo trimestre da gravidez, Ultrassonografia pré-natal/métodos, Reprodutibilidade dos testes

## Abstract

**Objective:**

The purpose of this study was to evaluate the agreement between and reproducibility of specific two-dimensional (2D) and three-dimensional (3D) ultrasound methods in assessing cervical volume in pregnant women in their second trimester.

**Materials and Methods:**

This was a prospective cross-sectional study of 48 asymptomatic pregnant women at 20–24 weeks of gestation. All cervical volumes were determined by transvaginal ultrasound, with a 2D method employing a geometric formula {π * [(anteroposterior diameter + transverse diameter) * ¼] * length}, where π = 3.14, and a 3D method employing a virtual organ computer-aided analysis. Intraobserver and interobserver reliability was analyzed by calculating the intraclass correlation coefficient (ICC).

**Results:**

The mean maternal age and timing of the ultrasound examination were 26 ± 6 years and 21 ± 1 weeks of gestation, respectively. The mean cervical volumes measured by the 2D and 3D ultrasound methods were 27.71 ± 9.27 cm^3^ and 35.21 ± 8.85 cm^3^, respectively. Cervical length and volume showed a positive correlation with both methods–r = 0.77 (*p* < 0.001) and r = 0.70 (*p* < 0.001), respectively. Intraobserver reliability was excellent for both methods, with ICCs of 0.92 and 0.93 for the 2D and 3D methods, respectively. Interobserver reliability was good (ICC: 0.81) for the 3D method, whereas it was poor (ICC: 0.37) for the 2D method. Reproducibility of the transverse diameter measurement was low, with an intraobserver ICC of 0.41 and an interobserver ICC of 0.48.

**Conclusion:**

Cervical volume measurements obtained with 2D and 3D ultrasound methods seem to show satisfactory agreement and good intraobserver reliability. In our study sample, the 2D ultrasound method showed low interobserver reliability, whereas the 3D ultrasound method was more reliable, with good intraobserver and interobserver reliability.

## Abstract (pt)

**Objetivo:**

O objetivo deste estudo foi avaliar a concordância e a reprodutibilidade dos métodos de ultrassonografia bidimensional (2D) e tridimensional (3D) na avaliação do volume cervical em gestantes no segundo trimestre.

**Materiais e Métodos:**

Foi realizado um estudo transversal prospectivo com 48 gestantes assintomáticas entre 20 e 24 semanas. Todos os volumes cervicais foram realizados pela ultrassonografia transvaginal utilizando os métodos 2D {π * [(anteroposterior + transverso) * ¼] * comprimento}, onde π = 3,14, e 3D (análise auxiliada por computador de órgão virtual). A confiabilidade intraobservador e interobservador foi analisada usando o coeficiente de correlação intraclasse (CCI).

**Resultados:**

A idade materna média e o momento do exame ultrassonográfico foram de 26 ± 6 anos e 21 ± 1 semanas, respectivamente. Os volumes cervicais médios medidos pelos métodos de ultrassonografia 2D e 3D foram de 27,71 ± 9,27 cm^3^ e 35,21 ± 8,85 cm^3^, respectivamente. O comprimento e o volume cervical apresentaram correlação positiva com os métodos 2D e 3D, com r = 0,77 (*p* < 0,001) e r = 0,70 (*p* < 0,001), respectivamente. A concordância intraobservador foi excelente para ambos os métodos, com CCI de 0,93 para o método 3D e 0,92 para o método 2D. A concordância interobservador para 3D foi boa, com CCI de 0,81, entretanto, a reprodutibilidade interobservador para o método 2D foi ruim (CCI de 0,37). A reprodutibilidade da medida do diâmetro transversal foi baixa, com CCI intraobservador de 0,41 e CCI interobservador de 0,48.

**Conclusão:**

As medidas do volume cervical obtidas com os métodos de ultrassonografia 3D e 2D apresentaram concordância satisfatória e boa reprodutibilidade intraobservador. O método 2D apresentou baixa reprodutibilidade interobservador. O método 3D foi mais confiável, com boa reprodutibilidade intraobservador e interobservador.

## INTRODUCTION

Preterm birth is a major cause of neonatal morbidity and mortality, accounting for more than half of all neonatal deaths^(^1^, ^2^)^. It is estimated that more than 15 million pregnancies worldwide culminate in preterm birth each year; therefore, identifying pregnancies that are at high risk for preterm birth and implementing effective interventions to prevent this outcome continue to be important issues in obstetrics^(^3^, ^4^)^.

Currently, transvaginal ultrasound is used in the second trimester of pregnancy to measure cervical length and assess the risk of spontaneous preterm birth^(^5^, ^6^)^, because of its high specificity (65–100% for a cervix ≤ 25 mm) and negative predictive value (86–97% for a cervix ≤ 25 mm). However, this parameter represents only one element of the overall process of cervical remodeling that precedes labor^(^7^, ^8^)^. Although there is no consensus on the cutoff point that defines a truly short cervix, its negative predictive value is high, whereas its sensitivity and positive predictive value are low^(^9, 10^)^. Data from the literature show that the positive predictive value of cervical length ranges from 6% to 44%, which means that most women who evolve to spontaneous preterm birth are not identified when their risk is assessed by cervical length alone^(^11^)^.

Measurement of cervical volume has been described in the literature as a new parameter for identifying pregnancies at risk of spontaneous preterm birth, and many studies have described this association as an inverse proportional relationship^(^12^, ^13^, ^14^)^. However, those studies have employed a variety of methods to measure cervical volume^(^15^, ^16^, ^17^)^, including two-dimensional (2D) ultrasound using geometric formulas and three-dimensional (3D) ultrasound using different types of volume analysis such as multiplanar and virtual organ computer-aided analysis (VOCAL). To our knowledge, there has been only one study that compared 2D (geometric formula-based) and 3D (VOCAL) ultrasound methods in the measurement of cervical volume^(^18^)^.

The purpose of this study was to evaluate the agreement between and reproducibility of specific 2D and 3D ultrasound methods in assessing cervical volume in pregnant women in their second trimester.

## MATERIALS AND METHODS

This was a prospective cross-sectional study of consecutive asymptomatic pregnant women with intact ovular membranes at 20–24 weeks of gestation, evaluated between July 2017 and January 2018. The exclusion criteria were the absence of uterine contractions and the presence of vaginal bleeding. Of the 52 women who were initially eligible for inclusion, four were excluded because they presented with vaginal bleeding. Therefore, the final sample comprised 48 pregnant women, all of whom underwent a second trimester ultrasound scan and transvaginal measurement of cervical length in the Department of Fetal

Medicine of the Fernandes Figueira National Institute of Women’s, Children’s and Adolescents’ Health, operated by the Oswaldo Cruz Foundation in the city of Rio de Janeiro, Brazil. Gestational age was confirmed by first trimester ultrasound with measurement of crown-rump length. This study was approved by the ethics committee of the institution (Reference no. 85093071.6.0000.5269), and all participants gave written informed consent.

The ultrasound examinations were performed with one of two comparable ultrasound systems (Voluson E6 or Voluson S8; GE Medical Systems, Zipf, Austria) devices equipped with an endocavitary volume transducer (RIC 5–9D; GE Medical Systems). The transvaginal examination was performed with the subject in the lithotomy position and with an empty bladder. The probe was inserted into the anterior fornix of the vagina, allowing the cervix and its midsagittal plane to be identified. The cervical length was measured between the internal and external os^(^19^, ^20^)^. The system was then switched to 3D mode (setting: Qual. high 1), and the image was adjusted so that the acquisition area included the entire cervix (internal os, external os, and lateral boundaries); the acquisition angle ranged from 86° to 140° according to the characteristics of the cervix. Finally, the automatic acquisition mode was activated with a scan angle of 60°. The acquired images were stored on the hard disk of the ultrasound machine for subsequent ana lysis. All volume datasets were acquired by the main examiner (observer 1), who had three years of experience in 3D ultrasound for cervical volume measurements.

With the 2D ultrasound method, the cervical length was measured in the midsagittal plane as a straight line between the internal and external os. The anteroposterior diameter was also measured in the midsagittal plane, and the width was measured in the axial plane. The caliper was positioned at the junction of the cervical margins. A geometric formula was then used to calculate the cylinder volume:

$$V=\pi {\left(AP+T\right)}^{\frac{1}{4}}\,length$$

where *V* is the volume, n = 3.14, *AP* is the anteroposterior (diameter), and *T* is the transverse (diameter).

**Figure 1:**
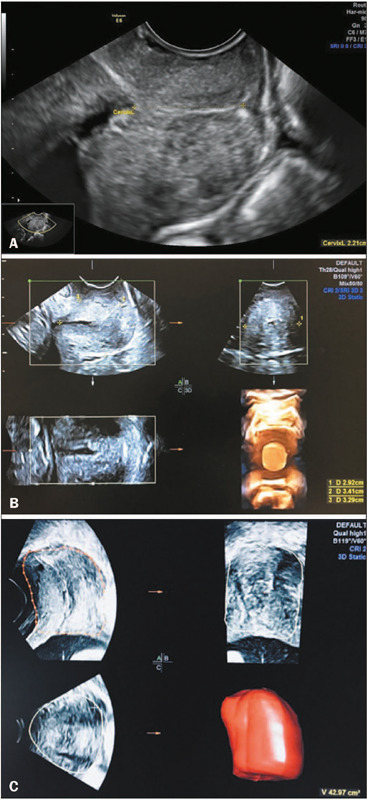
**A:** Cervical length measurement In the m¡dsag¡ttal plane between the internal and external os. **B:** Cervical measurement of the length anteroposterior diameter, and transverse diameter in the sagittal and axial planes (A and B, respectively) to calculate volume using the 2D ultrasound method. **C:** Cervical volume using the 3D (VOCAL) ultrasound method with a 30° angle of rotation and manual delineation of the cervical surface using the axial (A) plane as the reference.

For 3D ultrasound method, the VOCAL technique was used, with the image being adjusted to align the cervical canal and with the longitudinal axis of rotation, as well as the gain, being adjusted to obtain the best identification of the cervical contours. The midsagittal plane was used as the reference, with manual segmentation and a rotation angle of 30°. The external surface of the cervix was outlined in six consecutive and adjacent planes; after outlining the last area, the software automatically calculated the volume with its 3D reconstruction (Figure 1).

Two observers, working independently, applied the 2D ultrasound method (measurements of the cervical length, anteroposterior diameter, and transverse diameter) and the 3D ultrasound method (VOCAL) to the same volume datasets. For each of the 55 volume datasets, observer 1 performed two cervical volume measurements using the 2D method and two using the 3D method, for a total of 220 measurements. To avoid recall bias, the second 2D and 3D measurements were made at least 7 days after the first measurements and the examiner was blinded to the first measurements. The second examiner (observer 2), who had five years of experience in 3D ultrasound for cervical volume measurements, performed only one set of cervical volume measurements using the 2D and 3D methods on each of the same volume datasets, for a total of 110 measurements. Each observer was blinded to the results of the other.

### Statistical analysis

The statistical analysis was performed with the Statistical Package for the Social Sciences, version 13.0 (SPSS Inc., Chicago, IL, USA). The Kolmogorov-Smirnov test was used in order to assess the normality of the distribution of the variables. Pearson’s correlation coefficient (r) was calculated to quantify the strength of the association between cervical length and cervical volume calculated by each of the two methods employed. Interobserver and intraobserver reliability was analyzed by determining the intraclass correlation coefficient (ICC). Bland-Altman plots were created to compare the mean difference between the methods. Values of *p* < 0.05 were considered statistically significant.

## RESULTS

A total of 48 pregnant women were evaluated with 2D and 3D transvaginal ultrasound. After the initial selection process, no cases were excluded, and there were no cases of cervical funneling or dilation of the cervix. Of the 48 women evaluated, 21 (43.8%) were nulliparous. The mean maternal age was 26 ± 6 years, and the mean gestational age at the time of the ultrasound examination was 21 ± 1 weeks (Table 1).

**Table 1:** Sociodemographic characteristics of the studied population.

Characteristic	(N = 48)
Maternal age (years), mean ± SD	26 ± 6
Gestational age (weeks), mean ± SD	21 ± 1
Body mass index (kg/m^2^)	
Mean ± SD	25.85 ± 6.13
< 18.5, n (%)	2 (4.2)
18.5–24.9, n (%)	23 (47.9)
25.0–29.9, n (%)	15 (31.2)
30.0–34.9, n (%)	4 (8.3)
35.0–39.9, n (%)	3 (6.2)
≥ 40.0, n (%)	1 (2.1)
Years of schooling	
Mean ± SD	10 ± 3
0–4, n (%)	3 (6.2)
5–9, n (%)	17 (35.4)
10–12, n (%)	19 (39.6)
≥ 13, n (%)	9 (18.8)
Ethnicity n (%)	
White	12 (25.0)
Mixed	24 (50.0)
Black	7 (14.6)
Other	5 (10.4)

SD, standard deviation.

Cervical volume data showed a normal distribution for both methods. The mean cervical volume measured by the 2D and 3D methods was 27.71 ± 9.27 cm^3^ and 35.21 ± 8.85 cm^3^, respectively (Table 2). The median, 10th percentile, and 5th percentile cervical volumes were, respectively, 35.56 cm^3^, 23.75 cm^3^, and 20.82 cm^3^ when measured by the 3D method, and 26.48 cm^3^, 16.86 cm^3^, and 14.89 cm^3^ when measured by the 2D method. Cervical length data also showed a normal distribution, with a mean of 3.5 ± 0.64 cm. Cervical length and volume showed a positive correlation with the 2D and 3D methods–r = 0.77 (*p* < 0.001) and r = 0.70 (*p* < 0.001), respectively.

**Table 2:** Descriptive analysis of cervical volume measurements by 2D and 3D ultrasound methods.

Measure	Mean ± SD	Variation
Cervical dimensions (cm)		
Length	3.5 ± 0.64	2.27 – 5.24
Anteroposterior diameter	3.12 ± 0.39	2.24 – 4.24
Transverse diameter	3.15 ± 0.36	2.06 – 4.40
Cervical volume (cm^3^)		
3D ultrasound method		
Observer 1		
1st measurement	32.95 ± 7.94	13.84 – 48.95
2nd measurement	34.22 ± 8.40	12.52 – 50.70
Observer 2		
Single measurement	38.44 ± 9.38	16.59 – 58.91
2D ultrasound method		
Observer 1		
1st measurement	24.80 ± 6.44	12.30 – 36.95
2nd measurement	23.02 ± 5.64	11.23 – 33.88
Observer 2		
Single measurement	35.30 ± 9.93	16.74 – 67.19

SD, standard deviation.

**Figure 2:**
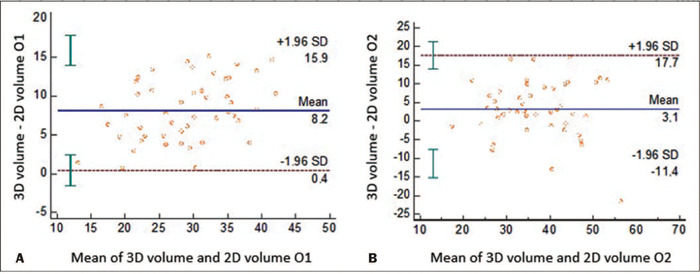
Bland-Altman plots of the mean differences between the 2D and 3D ultrasound methods for measuring cervical volume and the 95% limits of agreement, for observer 1 (O1, in **A)** and observer 2 (O2, in **B).**

**Figure 3:**
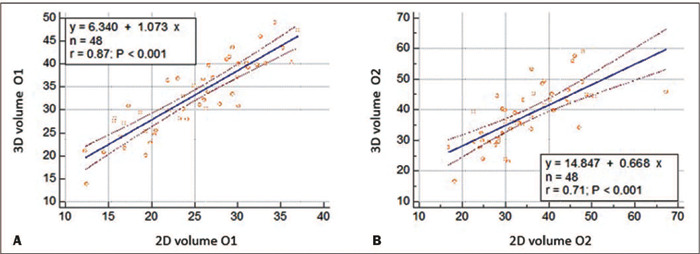
Linear regression graphs showing the correlation between the cervical volume measurements obtained with 2D and 3D ultrasound methods by observer 1 (O1, in **A)** and observer 2 (O2, in **B).**

The agreement between the 2D and 3D ultrasound methods was considered acceptable. The Bland-Altman plot in Figure 2 shows the mean differences in the calculated volumes and the limits of agreement between the two methods in terms of the cervical volume data. Figure 3 shows the positive correlation between the two methods for cervical volume, with r = 0.87 for observer 1 and r = 0.71 for observer 2 (*p* < 0.001). For observer 1, the ICC between the methods was 0.58 (95% confidence interval [CI]: 0.25 to 0.76), whereas it was 0.80 (95% CI: 0.64 to 0.88) for observer 2 (Table 3).

**Table 3:** Reliability analysis of cervical volume measurements by 2D and 3D ultrasound methods.

Measure	ICC	95% CI
Inter-method reliability		
Observer 1	0.58	0.25 to 0.76
Observer 2	0.80	0.64 to 0.88
Interobserver reliability		
3D ultrasound method	0.81	0.67 to 0.89
2D ultrasound method	0.37	−0.10 to 0.65
Intraobserver reliability		
Observer 1		
3D ultrasound method	0.93	0.88 to 0.96
2D ultrasound method	0.92	0.87 to 0.96

**Figure 4:**
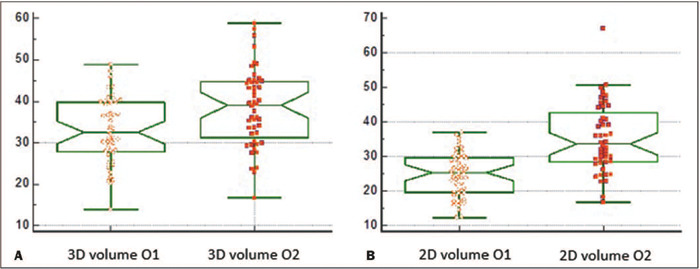
Box plots comparing cervical volume measurements obtained with 3D and 2D ultrasound methods **(A** and **B,** respectively) by observer 1 (O1) and observer 2 (O2).

Table 3 shows the results of the intraobserver and in-terobserver reliability analysis for each ultrasound method. We can see that the intraobserver reliability was excellent for the 2D and 3D methods, based on the high ICC values: 0.92 (95% CI: 0.87 to 0.96) and 0.93 (95% CI: 0.88 to 0.96), respectively. As illustrated in Figure 4, interobserver reliability was good for the 3D method (ICC: 0.81; 95% CI: 0.67 to 0.89), whereas it was poor for the 2D method (ICC: 0.37; 95% CI: -0.10 to 0.65).

The results of the analysis of agreement of the 2D measurements of the cervix (length, anteroposterior diameter, and transverse diameter) are shown in Table 4. The most relevant finding of this analysis is the low reproducibility of the transverse diameter measurement, with an intraobserver ICC of 0.41 (95% CI: −0.04 to 0.67) and an interobserver ICC of 0.48 (95% CI: 0.08 to 0.70).

**Table 4:** Reliability analysis of cervical measurements by the 2D ultrasound method.

Measure	ICC	95% CI
Intraobserver reliability		
Observer 1		
Length	0.95	0.91 to 0.97
Anteroposterior diameter	0.94	0.89 to 0.96
Transverse diameter	0.41	−0.04 to 0.67
Interobserver reliability		
Observer 1 vs. observer 2		
Length	0.60	0.30 to 0.78
Anteroposterior diameter	0.57	0.25 to 0.76
Transverse diameter	0.48	0.08 to 0.70

## DISCUSSION

Although the assessment of cervical volume during pregnancy is still primarily used in scientific research, some authors suggest that this measurement may have clinical significance^(^9^, ^15^)^. In clinical practice, the measurement of cervical length is the best described, most standardized technique and is an important tool for predicting spontaneous preterm birth^(^19^, ^20^, ^21^)^. Nevertheless, that measurement has some limitations due to its low sensitivity and positive predictive value, which are associated with the low prevalence of a short cervix in low-risk pregnant women^(^22^, ^23^)^. Therefore, the development of a complementary tool to predict the risk of preterm birth has motivated the search for a better understanding of the volume of the cervix during pregnancy, although the best way to measure this volume is still unclear.

In the present study, we compared cervical volume measurements using the 3D ultrasound method with VOCAL software and the 2D ultrasound method that employs the geometric formula for cylinders. The main finding was the high intraobserver and interobserver reliability for measurements made with the 3D ultrasound method, as well as the high intraobserver reliability for measurements made with the 2D ultrasound method. The cervical volumes calculated by both methods showed good reliability and correlated positively between the two observers.

Rojas et al.^(^24^)^ evaluated 32 pregnant women who underwent transvaginal ultrasound, assessing the reproducibility of cervical volume measured by 3D ultrasound with the VOCAL technique, with 30° rotation and manual segmentation. The authors observed values of ICC > 0.90 for intraobserver and interobserver reliability. In another study, Basgul et al.^(^25^)^ evaluated 126 pregnant women by 3D transvaginal ultrasound using the VOCAL technique for the cervical volume assessment. The authors observed good intraobserver and interobserver reliability, with an ICC of 0.95 for both.

In the present study, interobserver reliability was poor for the cervical volume measurements assessed by 2D ultrasound, which can be attributed to the low interobserver reliability of the transverse diameter measurements, which can in turn be attributed to the well-reported difficulty in correctly assessing the boundary between the cervix and the lateral vaginal wall in the transverse plane of the cervix^(^26^, ^27^)^. However, in a study on the reproducibility of cervical length and width measurements during pregnancy, 20 women were analyzed at 15–37 weeks of gestation by two observers, who each made three measurements of each parameter, and the authors found good reproducibility for the measurement of the transverse diameter^(^28^)^.

When we compared the 2D and 3D ultrasound measurements of cervical volume in our study, we found that the volumes determined by the 3D method were larger than those determined by the 2D method. Ahmed et al.^(^18^)^ studied 142 pregnant women at high risk for preterm birth, evaluated at 16–24 weeks of gestation, assessing cervical volume with 3D and 2D ultrasound methods (VOCAL and geometric formula-based methods, respectively). Those authors also found that cervical volume was lower with the 2D method than with the 3D method. In addition, they found that, although both methods had good reproducibility, the limits of agreement were wider when the 3D method was applied. The VOCAL technique tends to overestimate volume calculation and is technically difficult to perform when applied to structures with poorly defined contours during rotation^(^29^)^, which may explain the higher cervical volume measurements found with the 3D ultrasound method. In an *in vitro* study, the interobserver reliability and validity of two 3D methods for volume assessment (multiplanar and VOCAL) were assessed and both were found to be highly reliable and valid to within 4% of the true volumes^(^30^)^. In that study, measurements made with a 6° rotation were significantly more valid than were those made with a 30° rotation or with the multiplanar technique.

Our study has some limitations. The main limitation is that the accuracy of cervical volume measurement in pregnancy cannot be assessed *in vivo.* Other limitations include the small sample size and the fact that we did not evaluate gestational or perinatal outcomes, given that it was a cross-sectional study. However, its strengths include an adequate statistical methodology to assess interob-server and inter-method reproducibility with pretrained observers.

## CONCLUSION

In summary, cervical volume measurements obtained with 3D and 2D ultrasound methods appear to show satisfactory agreement and good intraobserver reliability. The 2D ultrasound method evaluated here showed low interobserver reliability, and the 3D method was more reliable, with good intraobserver and interobserver reliability.
